# Nursing students’ experiences in virtual simulation teaching: a meta-synthesis of qualitative research

**DOI:** 10.3389/fpubh.2026.1863553

**Published:** 2026-06-22

**Authors:** Guoyong Zhang, Xianghong Sun, Dan Yang, Qinwen Shao

**Affiliations:** 1 School of Medical Management, Shandong First Medical University, Jinan, China; 2 Second Intensive Care Unit, Shengjing Hospital of China Medical University, Shenyang, China; 3 School of Medicine and Pharmacy, Wuhan University of Bioengineering, Wuhan, Hubei, China

**Keywords:** experiences, meta-synthesis, nursing students, virtual reality, virtual simulation

## Abstract

**Aim:**

This study is aimed to explore nursing students’ experiences in virtual simulation (VS) teaching and further identify the challenges and needs they encounter.

**Design:**

A qualitative meta-synthesis.

**Methods:**

Nine databases including PubMed, Web of Science, CINAHL, Embase, Cochrane Library, CNKI, Wangfang Data, VIP, and CBM were searched from each database’s inception to February 7, 2025. Studies were critically appraised using the Joanna Briggs Institute (JBI) Critical Appraisal Checklist for Qualitative Research. Qualitative data were extracted, summarized, and meta-synthesized.

**Results:**

A total of 17 research studies from eight countries were included in the meta-synthesis. Sixty-five main themes and 156 sub-themes were extracted, which were eventually integrated into nine categories and four themes: (1) emotional experiences; (2) advantages of VS teaching; (3) challenges of VS teaching; (4) students’ needs for VS teaching.

**Conclusion:**

VS technology demonstrates significant advantages in enhancing clinical practice skills. However, future development of VS devices should prioritize simplifying interactive designs to lower operational barriers and explore multimodal feedback mechanisms to improve the realism of virtual patients. Additionally, developing case libraries with diverse scenarios and multilingual support to enhance the richness and practicality of teaching.

## 1 Introduction

Thanks to the development of information technology, teaching methods in nursing education are continuously innovating to meet the clinical demand for high comprehensive abilities in nursing students. Over the past decade, the application of virtual simulation (VS) in nursing education has significantly increased (1). VS is defined as an interactive educational process in which users participate in clinical scenarios through a computer screen, the internet or a digital learning environment (2). Currently, there are three commonly used VS technologies in the field of healthcare education and practice: haptic device simulators, computer-based simulations, and head-mounted displays (HMDs) (3). Within the diverse array of technologies employed in VS, virtual reality (VR) stands out and is attracting growing interest, thanks to the profound sense of immersion it delivers. VR technology is defined as “a broad class of computer applications characterized by immersion, high visualization, and three-dimensional features, allowing participants to explore and navigate through seemingly real or physical environments” (4). The two more immersive technologies, haptic device simulators and head-mounted displays (HMDs), are referred to as VR technology. However, many studies do not distinguish between VS and VR (5, 6). Characterized by immersion, interactivity, and imaginative potential, VR recreates authentic clinical environments through computer technology, eliminating risks of operational errors and patient safety hazards (3). The superiority of VR over traditional practical teaching methods has been demonstrated (7). In recent years, an increasing number of scholars have focused on students’ experiences with VR teaching (8–10), as these perspectives are critical for developing student-centered VR teaching devices in the future. Existing studies indicate that students’ experiences with VR teaching are complex (10, 11), yet no systematic review has synthesized these experiences.

## 2 Background

Since the COVID-19 pandemic in 2019, the application of VS especially VR in nursing education has expanded significantly (12–14). Current research primarily explores the effects of VS across diverse practical training programs. Studies show significant outcomes in VS-based training for oral care (13), nasogastric tube feeding (14), and disaster response (15). VS not only enhances nursing students’ technical proficiency but also improves their clinical reasoning skills (16), problem-solving abilities, self-efficacy (17), and learning satisfaction (7). However, some reports highlight side effects of VR learning, such as simulator sickness, including symptoms like nausea, dizziness, or eye strain (18), though these issues are typically mild or rare. As VS gains widespread adoption in nursing education, more scholars are examining students’ experiences with VS teaching (8–10). The majority of students express strong interest in VS-based learning, especially in VR, describing it as novel and engaging (6, 19). VR allows them to practice skills repeatedly in rich clinical scenarios, substantially improving learning outcomes. Nevertheless, some students have critiqued VR’s limitations, such as insufficient realism or operational complexity (19). Compared with VR, general VS systems (computer-based simulations) are slightly inferior in terms of immersion and interactivity. However, they can still provide students with relatively realistic clinical scenarios for practice skills training, which greatly piques their interest. Compared with traditional experimental teaching, VS is more effective in improving students’ knowledge mastery and clinical reasoning abilities, while also enhancing the satisfaction of nursing students with their learning experience (20). Nevertheless, based on their experience, students have indicated that the VS system provides insufficient timely feedback (9) and needs further refinement. Overall, students’ experiences with VS teaching are multifaceted, varying by individual backgrounds and potentially influenced by differences in VR devices used. Integrating these experiences is essential, yet no literature has synthesized this complex landscape of student learning experiences. This study conducts a systematic review of qualitative research on students’ participation in VS teaching, aiming to understand challenges and needs encountered in VS learning. The findings will provide theoretical foundations for optimizing VS teaching equipment and accommodating diverse cultural backgrounds and learning foundations in future educational design.

## 3 Review

### 3.1 Aim

This study aims to synthesize qualitative research on experiences and needs of nursing students in VS teaching, in order to provide references for improving instructional effectiveness.

### 3.2 Design

This research employed the meta-synthesis approach recommended by the Australian Joanna Briggs Institute (JBI) Evidence-Based Health Care Center (21). The process involved collecting categories and themes from the included studies, and classifying them based on theme similarity. Through continuous comparison and thematic analysis, comprehensive thematic findings were ultimately formed (21).

### 3.3 Methods

#### 3.3.1 Search strategy

According to the JBI guidelines (21), we initially conducted a preliminary and limited search in PubMed using the key words “nursing students,” “virtual simulation,” and “experience.” We determined the final search strategy by analyzing the key words in the titles and abstracts of identified articles. The search terms we finally settled on were as follows: (nursing student* OR nursing OR student* OR students, nursing) AND (VR OR VS OR VR simulation) AND (qualitative research OR qualitative OR phenomeno* OR grounded theory OR ethnography OR experience* OR feeling* OR perception* OR attitude* OR perspective* OR opinion* OR emotion*). The search fields were Title/Abstract and MeSH terms. A systematic search was conducted using these terms in both Chinese and English databases, including PubMed, Web of Science, CINAHL, Embase, Cochrane Library, CNKI, Wangfang Data, VIP, and Sinomed. The search time frame covered the period from inception to February 7, 2025, with a language limit of Chinese and English. In order to avoid missing eligible studies, the reference lists of all included articles were searched to find additional studies. The search strategy on PubMed is shown in Supplementary material 1.

#### 3.3.2 Inclusion/exclusion criteria

The inclusion criteria were formulated based on the PICoS (Participants, Phenomenon of interest, Context, Study design). Detailed inclusion and exclusion criteria are specified in Table 1. After importing database-retrieved records into EndNote for deduplication, two researchers independently conducted title/abstract review to assess eligibility, constituting the initial screening phase. Studies that passed this preliminary assessment, full-text evaluation followed to verify compliance with inclusion criteria, while excluding review articles and off-topic research. A secondary screening was subsequently performed, with qualified studies proceeding to final inclusion in this analysis.

**Table 1:** Inclusion/exclusion criteria.

Inclusion criteria	Exclusion criteria
P(Participants): nursing students or participants included nursing students.	Unavailable full-text research
I(Phenomenon of Interest): the feelings and experiences of nursing students participating in VS teaching. VS technology includes haptic device simulators, computer-based simulations, and head-mounted displays (HMDs).	Incomplete information in the research
Co(Context): In the VS experiment teaching environment of nursing students.	Research not related to the topic
S(Study design): Qualitative research using phenomenology, descriptive research, grounded theory, ethnography, and other research methods.	Research not written in Chinese or English

#### 3.3.3 Quality appraisal

JBI Critical Appraisal Checklist for Qualitative Research (21) was used for quality appraisal of included literature. This tool includes 10 quality criteria: (1) the philosophical perspective and the research methodology; (2) the research methodology and the questions; (3) the research methodology and the data collection methods; (4) the research methodology and the data analysis; (5) the research methodology and the interpretation of the results; (6) the description of the culture or the theoretical grounding of the researchers; (7) the influence of the researchers on the research; (8) the representativeness of the participants’ voices; (9) the proof of ethical approval of the study; (10) the relationship between the conclusions and results. Each evaluation item has three evaluation results: yes, no, and not applicable. If all evaluation criteria were met, the article’s bias risk was considered to be low, which would be graded A. If partial evaluation criteria were met, the article’s evaluation rating was B. If all evaluation criteria were not met, the article’s bias risk was considered to be high, with an evaluation rating of C. The quality assessment was independently performed by two researchers, with disagreements resolved through consultation with a third reviewer. Studies rated as A or B were included, while those graded C were excluded.

#### 3.3.4 Data abstraction

The information contained in the research studies was extracted using the JBI standardized data extraction tool for qualitative research. Specifically, the following details were retrieved: Author; year; country; aim; methodology; participants; data collection and data analysis; Results. This extraction process was performed independently by two researchers after repeated reading, with disagreements resolved through adjudication by a third researcher.

#### 3.3.5 Data synthesis

This study employed a meta-synthesis methodology endorsed by the Joanna Briggs Institute (JBI) Health Care Centers (21) to synthesize findings from qualitative research. Two independent reviewers conducted iterative readings of each included study to achieve data familiarization. Two reviewers collected the study results, including the themes, implied meanings and categories. Thereafter, they further synthesized and summarized the study results according to their meanings to ensure that they are targeted, persuasive and general. On the premise of understanding the philosophical thought and methodology of qualitative research, reviewers repeatedly read, analyzed and interpreted the previous study results. Next, they summarized and combined similar results to form the new genera and then integrated the new genera into the study results. The two reviewers discussed the disagreements until a consensus was reached. The findings extracted from the included research studies were integrated and classified based on their similarity in meaning, forming new categories. Two researchers further integrated these categories to form more comprehensive themes.

## 4 Results

### 4.1 Search outcome

**Figure 1:**
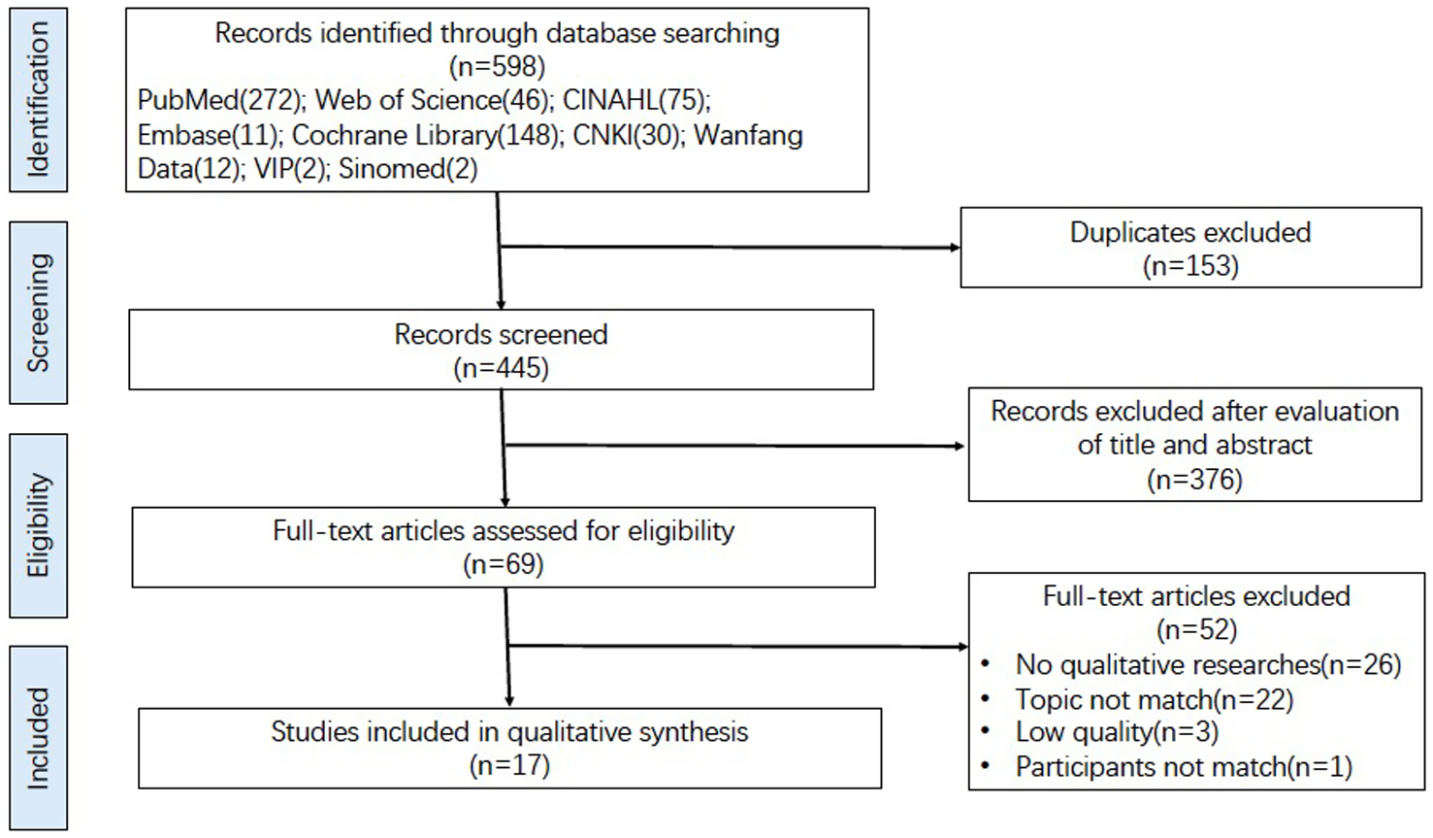
Flow diagram of literature selection.

After a systematic search of nine databases, 598 articles were obtained, 153 of which were duplicated. The remaining 445 papers were read titles and abstracts, and 376 papers that did not meet the inclusion criteria were excluded. Sixty-nine papers were read as full-text, while non-qualitative studies (*n* = 26), topics not matched (*n* = 22), low quality (*n* = 3), participants not matched (*n* = 1) were excluded. Finally, 17 research studies were included for further quality assessment. Figure 1 shows the flow diagram of the literature selection.

### 4.2 Quality appraisal

Quality appraisal was conducted on the 17 screened studies. All 17 articles received a quality rating of grade B, indicating possible bias. Despite this, the overall methodological quality of these 17 studies was considered high, and all were ultimately included in the subsequent meta-analysis process. The quality assessment results are presented in Table 2.

**Table 2:** Methodological quality appraisal of the included studies.

Study	Item1	Item2	Item3	Item4	Item5	Item6	Item7	Item8	Item9	Item10	Overall appraisal
Jing et al. (27), 2020	Y	Y	Y	Y	Y	N	N	Y	Y	Y	B
Saab et al. (18), 2021	Y	Y	Y	Y	Y	N	N	Y	Y	Y	B
Bing et al. (35), 2023	Y	Y	Y	Y	Y	N	N	Y	Y	Y	B
Joung and Kang (22), 2022	Y	Y	Y	Y	Y	N	N	Y	Y	Y	B
Singleton et al. (6), 2021	Y	Y	Y	Y	Y	N	N	Y	Y	Y	B
Mäkinen et a. (34), 2023	Y	Y	Y	Y	Y	N	N	Y	Y	Y	B
Helle et al. (10), 2023	Y	Y	Y	Y	Y	N	N	Y	Y	Y	B
Saab et al. (18), 2021	Y	Y	Y	Y	Y	N	N	Y	Y	Y	B
Lee et al. (36), 2024	Y	Y	Y	Y	Y	N	N	Y	Y	Y	B
Jeon et al. (5), 2020	Y	Y	Y	Y	Y	N	N	Y	Y	Y	B
Kim et al. (11), 2021	Y	Y	Y	Y	Y	N	N	Y	Y	Y	B
Gao and Zhu (9), 2023	Y	Y	Y	Y	Y	N	N	Y	U	Y	B
Calik et al. (24), 2024	Y	Y	Y	Y	Y	N	N	Y	U	Y	B
Chang and Lai (8), 2021	Y	Y	Y	Y	Y	N	N	Y	Y	Y	B
Yeo and Jang (25), 2023	Y	Y	Y	Y	Y	N	N	Y	Y	Y	B
Verkuyl et al. (26), 2017	Y	Y	Y	Y	Y	N	N	Y	Y	Y	B
Avşar et al. (23), 2024	Y	Y	Y	Y	Y	N	N	Y	Y	Y	B

Appraisal Checklist: ① Is there congruity between the stated philosophical perspective and the research methodology? ② Is there congruity between the research methodology and the research questions or objectives? ③ Is there congruity between the research methodology and the data collection methods? ④ Is there congruity between the research methodology and the representation and the data analysis? ⑤ Is there congruity between the research methodology and the interpretation of results? ⑥ Is there a statement locating the researcher culturally or theoretically? ⑦ Is the influence of the researcher on the research, and vice versa, addressed? ⑧ Are participants and their voices adequately represented? ⑨ Is the research ethical according to current criteria or, for recent studies, and is there evidence of ethical approval by an appropriate body? ⑩ Do the conclusions drawn from the research report flow from the analysis or interpretation of the data?

Appraisal result: “Y”, Yes; “N”, No; “U”, Unclear; “N/A”, Not applicable.

### 4.3 Study characteristics

The included research studies were published between 2017 and 2024, covering eight countries, including four from China, two from Ireland, five from South Korea, one from the UK, one from Finland, one from Norway, two from Turkey, and one from Canada. All 17 studies employed descriptive qualitative research methods, with semi-structured interviews being the primary data collection approach. Among them, six studies utilized focus group interviews, and three studies combined both semi-structured interviews and focus group methods. Among the 17 included research studies, 10 employed computer-based VS technology, while seven adopted more immersive VR technologies, including VR headsets, VR glasses, or haptic simulators. The characteristics of the included studies are presented in Table 3.

**Table 3:** Characteristics of the included studies.

Author, Year	Country	Aim	Methodology	Participants	Data collection/analysis	Results
Jing et al. (27), 2020	China	Exploring the experience and effectiveness of nursing undergraduate students using VS experiment teaching projects	Qualitative research	Nine nursing students	Semi-structured interviews/thematic content analysis	Four themes: (1) Expanding thinking, (2) The development of systems thinking, (3) Considering issues from a nurse’s perspective, and (4) Supplementing clinical thinking in real-world scenarios
Saab et al. (19), 2021	Ireland	This study explored nursing students’ views of using VR in healthcare	Qualitative study	26 nursing students	Semi-structured interview, focus groups/thematic analysis	Four themes: (1) Positive experiences of VR, (2) Challenges to using VR, (3) settings where VR can be implemented, and (4) blue-sky and future applications of VR
Bing et al. (35), 2023	China	To understand the real experiences of undergraduate nursing students with VS experimental teaching	Qualitative research	21 nursing students	Semi-structured interviews/thematic content analysis	Five themes: (1) There are disparities in nursing students’ perceptions of VS experimental teaching, (2) Nursing students exhibit a lack of readiness for VS experimental teaching, (3) External environmental factors influence nursing students’ cognitive attitudes toward VS experimental teaching, (4) VS experimental teaching increases the learning pressure on nursing students and demands a high level of self-directed learning ability, (5) Concentrated VS experimental practice is the preferred format among nursing students
Joung and Kang (22), 2022	Korea	To explore the experiences of psychiatric nursing students in South Korea who participated in VS-based education during the COVID-19 pandemic	Qualitative study	20 nursing students	Focus-group interviews/Inductive content analysis	Three themes: (1) Glad that the patients were not real people, (2) Bridge between the text world and the real world, (3) Supplementations needed for VSs to replace clinical practice
Singleton et al. (6), 2021	United Kingdom	To explore nursing students’, simulation technicians’, and lecturers’ experiences of using a VR simulation to support learning about the recognition and management of an acute diabetic emergency	Descriptive qualitative study	21 staff and students	Focus group discussions, semi-structured interview/thematic analyses	Five themes: (1) Engagement, (2) Immersion, (3) Confidence, (4) Knowledge, (5) Challenges
Mäkinen et al. (34), 2023	Finland	To describe nursing students’ user experiences’ (UX) regarding highly immersive VR simulation with head-mounted display used for learning	Qualitative descriptive study	41 nursing students	Semi-structured interviews/deductive and inductive content analysis	Three themes: (1) Nursing care in the immersive VR simulation, (2) Technology in the immersive VR simulation, (3) Learning nursing in the immersive VR simulation
Helle et al. (10), 2023	Norway	To explore occupational therapy, social education, nursing, and social work students’ experiences with VR simulation as a learning activity in an interdisciplinary subject	Qualitative study	28 students (occupational therapy, social education, nursing, social work)	Semi-structured focus groups/thematic analysis	Three themes: (1) 360° videos provide observations for individual learning, (2) 360° videos activate emotional learning, (3) Debrief sessions enhance comprehensive learning
Saab et al. (18), 2021	Ireland	To explore nursing students’ perspectives of incorporating VR in nurse education	Qualitative descriptive study	26 nursing students	Semi-structured interview, focus groups/thematic analysis	Three themes: (1) Captivating, innovative, and empowering nature of VR, (2) Contextual transfer, (3) Challenges and threats to actualization
Lee et al. (36), 2024	Korea	To explore the experiences of nursing students whose clinical practice in mental health nursing had been substituted with VS programs due to the COVID-19 pandemic	Qualitative study	10 nursing students	Individual interviews/thematic analysis	Five themes: (1) Lack of vibrancy in the actual clinical setting, (2) Limited direct and indirect practical experience, (3) Performing diverse roles in a virtual setting, (4) Learner-directed practicum, (5) Sense of relief due to a safe virtual practicum environment
Jeon et al. (5), 2020	Korea	To explore the essential components and improvements needed in an adult nursing VR-based simulation training program for nursing students	Qualitative study	14 nursing students	Focus group interviews/Colaizzi’s phenomenological methodology	Four themes: (1) Limitations of clinical practice, (2) Benefits of simulation training, (3) Need to improve simulation training, (4) Need for VR-based simulation training
Kim et al. (11), 2021	Korea	To understand prelicensure nursing students’ perceptions and experiences of using VS as an alternative to clinical practice during the coronavirus 2019 (COVID-19) pandemic in South Korea	Descriptive qualitative study	20 nursing students	Focus group interviews/inductive content analysis	Three themes: (1) Difficulties encountered in using VS, (2) Benefits to student confidence and competence to provide patient-centered care, (3) Gaps in satisfaction due to needed improvements
Gao and Zhu (9), 2023	China	To explore learning experience of virtual simulation class experiment teaching and learning based on the perspective of nursing students	Qualitative study	14 nursing students	Semi-structured interview/the Colaizzi seven-step analysis	Two themes: (1) Positive experiences: a. The scene is real and diverse, with deep memory points, b. VS experiments can break the time, space, and location limitations, c. The display of knowledge is more logical, visualized and Stereoscopic, d. This is a popular trend; (2) Negative experiences: a. Learning efficiency varies by grade and subject, b. Inability to receive feedback in time for learning reflections
Calik et al. (24), 2024	Turkey	To explore the views of nursing graduate students about their experience with smart glasses	Qualitative study	13 nursing students	Semi-structured interview/thematic analysis	Five themes: (1) Emotional experiences, (2) Positive experiences, (3) Disadvantages of smart glasses, (4) Future applications of smart glasses, (5) Opinions on game design
Chang and Lai (8), 2021	Taiwan, China	To understand the experience of nursing students in using VR skill learning process	Qualitative study	60 nursing students	Focus group interviews/content analysis	Five themes: (1) Convenient to practice, but requires adaptation, (2) Fast skill learning process, (3) Stress-free learning environment, (4) Environmentally friendly, (5) Lacks a sense of reality
Yeo and Jang (25), 2023	Korea	To explore nursing students’ self-directed problem-solving in web-based VS experiences	Qualitative study	16 nursing students	Semi-structured interview/thematic analysis	Four themes: (1) Self-awareness of a lack of nursing competency in VR, (2) Applying new learning strategies learned from failure, (3) Voluntary learning behavior, (4) Cognitive shift toward a holistic understanding
Verkuyl et al. (26), 2017	Canada	To explore students’ experiences of the virtual gaming simulation	Qualitative study	20 nursing students	Focus group interviews/thematic analysis	Five themes: (1) Experiential learning, (2) The learning process, (3) Personal versus professional, (4) Self-efficacy, (5) Knowledge
Avşar et al. (23), 2024	Turkey	To examine nursing students’ experiences and perceptions regarding the ventrogluteal injection training provided through VR technology	Qualitative study	12 nursing students	Semi-structured interview/thematic analysis	Two themes: (1) Weaknesses of VS, (2) Strengths of VS

## 5 Meta-synthesis

A total of 65 themes (see “Results” in Table 3 for detailed information) and 156 sub-themes (see “Study findings” in Table 4 for detailed information) were extracted from the 17 included research studies. Two researchers read the full text carefully and extracted the information. After repeated comparison and classification, these themes were integrated into nine categories, resulting in four major synthesized findings: (1) Emotional experiences; (2) advantages of VS teaching; (3) challenges of VS teaching; (4) students’ needs for VS teaching. The specific synthesized process is presented in Table 4.

**Table 4:** Synthesized findings, categories and findings extracted from the included studies.

Synthesized findings	Categories	Study findings
Emotional experiences	Positive experiences	(1) Feel less psychological burden; (2) reduce fear; (3) boost confidence; (4) enjoyment; (5) less pressure, safer; (6) excited; (7) provide privacy; (8) reduce embarrassment
	Negative experiences	(1) Fear; (2) worried/anxious; (3) burnout
Advantages of VS teaching	Advantageous characteristics of VS	(1) Repeated practice is allowed and trial and error are permitted; (2) ease of use; (3) immersion/real; (4) interesting; (5) interaction; (6) safer; (7) convenient for practice; (8) save resources/time; (9) promote the fairness of practical teaching content
	VS teaching enhances students’ learning outcomes	(1) Improve knowledge/skill levels; (2) Reinforce learning; (3) personalized learning; (4) self-study and take the initiative to solve problems; (5) promote reflection; (6) cultivate critical thinking ability
	VS teaching deepens students’ professional cognition	(1) Enhancing practical comprehension of nursing roles; (2) enhance students’ sense of professional responsibility
Challenges of VS teaching	Deficiencies of VS	(1) Operation complex and requires adaptation/practice; (2) requires proficiency in English; (3) unable to experience the emotions of interacting with real people; (4) real touch sensation cannot be experienced; (5) scene is not rich enough, and the graphic quality and feedback are limited; (6) equipment resources are limited and cost is high; (7) cannot replace clinical practice training
	Challenges for students to using VS	(1) Adverse physical reactions: dizziness, nausea, vision problems; (2) risk of injury; (3) unable to adapt to different cultural/medical backgrounds; (4) inadequate self-ability results in failed response; (5) low participation
Students’ needs for VS teaching	VS technology require optimization	(1) enhance the diversity of scenarios; (2) improve realism and immersion; (3) design training content flexibly; (4) diversify the application languages
	VS teaching cases require optimization	(1) cases more diversified; (2) cases incorporate cultural contexts

### 5.1 Emotional experiences

Compared with traditional experimental teaching, VS teaching is a novel teaching format. Its immersion and interactivity make it engaging for students. The vast majority of students exhibit positive emotional experiences after experiencing VS teaching, while a minority demonstrate negative emotional responses following the simulation.

#### 5.1.1 Positive experiences

The vast majority of nursing students felt excitement after engaging in VS teaching. The highly immersive experimental scenarios provided a novel experience, which they found intriguing and enjoyable, with some even reveling in the process. In clinical practice, students often face pressure from both supervisors and patients during procedures. Some students fear causing harm to patients due to operational errors, leading to anxiety, while mistakes during procedures also embarrass and stress them. VS teaching effectively addresses these issues. Within highly realistic and safe virtual clinical settings, students can perform without fear of harming patients, reducing psychological burdens. Moreover, virtual experiments allow students to repeat practices after mistakes, protecting their privacy. This repetition hones their operational skills and boosts their confidence. As students said:

*‘I had a vague fear of working face-to-face with patients, but I was relieved to be able to experience in advance the interventions used in such situations through vSim.’* (22).

*‘…At first, I was very excited, then I realised that I could do something with the help of my teacher. My excitement was relieved, and I was able to do the steps slowly…’* (23).

#### 5.1.2 Negative experiences

However, a small portion of students reported that VS teaching induced feelings of fear and anxiety and burnout. These emotions primarily stemmed from the unfamiliarity with the unknown experiences presented by the virtual scenarios. Students’ lack of proficiency in VS technology led to concerns that their own clumsiness might result in operational errors. Prolonged VS learning gradually leads to student boredom.

*‘I am afraid of what can be done because I need a better grasp of new technologies.’* (24).

### 5.2 Advantages of virtual simulation teaching

In VS teaching, students have experienced the advantages of this instructional approach. The mechanism of the VS system, which allows trial and error and provides timely error-correcting feedback, further facilitates student reflection, enhances their knowledge and skill levels, and fosters the development of clinical thinking abilities and critical thinking capabilities.

#### 5.2.1 Advantages of virtual simulation

VS teaching exhibits unique advantages distinct from traditional experimental instruction. VS devices provide students with immersive and secure virtual clinical practice environments. These scenarios are intuitive, authentic, and easy to comprehend, enabling flexible operation. The system allows students to practice repeatedly on virtual patients, tolerates trial-and-error, and its error-correction feedback mechanism promotes reflective learning. Since virtual experiments do not consume medical supplies, they conserve resources to some extent and eliminate constraints related to teaching resource limitations, ensuring every student can fully engage in practice. This approach fosters equitable content access in practical training. A student expressed it like this:

*‘I realized that gaining in-depth experience and that repetition is really critical through vSim and that the knowledge gained from experience is internalized through procedural memory and makes it possible to react quickly to rapidly changing situations…’* (25).

*‘After completing vSim, the feedback turns up as an X mark. I see that X mark and rethink* *and check which aspects I had not fulfilled…’* (25).

#### 5.2.2 Virtual simulation teaching enhances students’ learning outcomes

The repetitive practice and timely error-correction feedback mechanisms of VS systems enhance students’ learning outcomes and accelerate the skill acquisition process. Within these educational systems, students can design personalized learning pathways based on their individual knowledge levels and skill proficiency. When encountering learning challenges, students are motivated to proactively resolve issues, while the system guides autonomous learning, thereby effectively enhancing both theoretical understanding and practical skills.

*‘Even when you made a mistake, you learn a lot more than choosing the right answer…* *you think,* ‘What am I doing wrong?’ *You think about it more and you remember it more.’* (26).

*‘…It gave you a choice of your actions, you can learn, then next time you can do it differently… I think it would help my learning because it would make me think about the options that might be available…’* (6).

#### 5.2.3 Virtual simulation teaching deepens students’ professional cognition

In authentic clinical scenarios, students begin to deliberate on operational strategies to address emergent conditions in simulated patients. Rather than merely executing procedural tasks mechanically, they approach clinical issues from the standpoint of prospective nurses. Students are required to respond with calmness and composure to fluctuations in patient conditions, conduct comprehensive assessments of intervention measures, and through continuous reflection, they gradually comprehend the essence of nursing. This process deepens their understanding of nursing practice and enhances their professional accountability.

*‘Upon entering the virtual simulation project, I immediately felt like a nurse with a professional responsibility to take good care of the patient, which naturally immersed me into the learning experience.’* (27).

*‘After entering the program, I approached every step with caution. Although it was a virtual environment, I worried that any mistake might negatively impact the patient, so I considered each detail thoroughly and anticipated potential issues from multiple perspectives.’* (27).

### 5.3 Challenges of virtual simulation teaching

Despite its unique advantages and popularity among students, VS teaching still faces several challenges. The first is the inherent limitations of the VS itself, and the second is the physical discomfort experienced by students during participation in these projects.

#### 5.3.1 Deficiencies of virtual simulation

The most frequently mentioned drawbacks of VS systems reported by students revolve around its perceived lack of “realism.” In virtual clinical settings, students feel unable to experience the emotional nuances of interacting with real human beings or the tactile sensations of patient contact, leading some to perform operations casually during training. Consequently, these students believe VS cannot replace hands-on clinical practice training. Additionally, some students noted that VS systems often involve complex operational procedures requiring extensive practice to master. The use of English in certain teaching systems poses a significant challenge for non-native English speakers. Furthermore, clinical cases within these systems, which are developed based on the cultural and medical backgrounds of their creators, can confuse students from diverse cultural contexts due to cultural discrepancies. Other reported issues include insufficient scenario diversity, low-quality graphics, unreasonable color schemes, and delayed feedback mechanisms in some commercially developed VS platforms. These technical shortcomings have also been highlighted by students. As students reported:

*‘…The models can be touched and felt, and therefore, they are realistic…’* (23).

*‘It was hard to accept [the VS program] because it was in English. It took a long time … because I am not that good at English. At first, [one scenario] took more than eight hours.’* (11).

#### 5.3.2 Challenges for students to using virtual simulation

Some students experienced adverse physical reactions after participating in VR teaching projects, such as nausea, dizziness, and vision problems, along with a risk of injury. Certain clinical cases within the system proved overly challenging, exceeding students’ abilities, leading to low engagement due to their inability to cope. Additionally, scenarios based on distinct cultural contexts created confusion for students due to cultural discrepancies. Issues like insufficient scenario diversity, poor graphics quality, unreasonable color schemes, and delayed feedback mechanisms in some systems have also been reported by students.

‘That happened to me as well! I was sweating up and then I felt a bit sick, but it went then after a while though, but during it, I did feel a bit sick, yes.’ (19).

### 5.4 Students’ needs for virtual simulation teaching

Despite certain limitations, the unique advantages of VS teaching position is as a valuable complement to clinical practice instruction. To enhance students’ learning experiences and improve the effectiveness of practical training, students maintain high expectations for future VS pedagogy while raising several key needs. These primarily revolve around optimizing VS technology and refining case scenarios.

#### 5.4.1 Virtual simulation technology requires optimization

Upgrading VS technology can significantly enhance students’ learning experiences. Increasing scenario diversity and refining details to create more realistic virtual environments will deepen student immersion. Students hope that VS systems can offer multilingual support instead of being limited to English. Additionally, considering varying foundational skills among students, they urge developers to design more flexible training content, enabling learners to better navigate scenarios according to their individual capabilities.

*‘Diverse scenarios can be used, so it will be fun’* (5).

*‘In regular simulation training, the model cannot realistically express higher levels of pain, such as sweating and shivering, and stuff, and we cannot observe these [signals]. Therefore, in VR, if the machine starts beeping or the patient evidently starts shivering or turns blueish, and if this emergency situation is expressed more realistically, would not we be able to better understand that, ‘oh, this is really an emergency?’* (5).

#### 5.4.2 Virtual simulation teaching cases require optimization

Students have also expressed demands regarding the cases within VS systems. They hope these cases can simulate a diverse range of clinical scenarios while taking into account users’ cultural backgrounds and language proficiency, ensuring easy comprehension for learners.

*‘I think it would be good if the simulation can provide an environment where there is a ward with 4–5 patients and you can [deliver] care for these patients as the nurse in charge of them.’* (5).

## 6 Discussion

This study aimed to synthesize existing research studies on nursing students’ experiences with VS teaching, aiming to clarify the challenges and needs encountered by students in this context. This review identifies four synthesized themes: (1) Emotional experiences; (2) advantages of VS teaching; (3) challenges of VS teaching; (4) students’ needs for VS teaching. Overall, students reported feeling excited and emotionally positive during VS-based learning. They described VS teaching as a novel and engaging instructional approach, with the majority expressing willingness to embrace this modality. Students acknowledged the educational advantages of VS, such as its immersive and interactive nature. However, they also highlighted limitations in current VS equipment and expressed hope for incremental improvements in future device development.

Nursing students exhibited dual emotional experiences toward VS teaching, including both positive and negative dimensions. Most students described predominantly positive emotions such as excitement, enjoyment, reduced stress, and confidence. These positive experiences enhanced their focus and engagement, subsequently improving knowledge and skill acquisition. This aligns with the research findings of Onah and colleagues (28), who posited that enjoyment and satisfaction experienced during learning serve as motivational factors driving learners’ sustained engagement. Motivational factors foster voluntary investment in learning among autonomous learners with high self-efficacy, while simultaneously cultivating interest and enjoyment toward tasks (29). Subsequent VS designs could enhance the effectiveness of VS teaching by triggering positive emotions, such as incorporating diverse and engaging plotlines or characters to spark students’ curiosity. Through real-time feedback and reward mechanisms, students’ sense of achievement can be strengthened, thereby motivating them to engage in more complex cognitive tasks. Conversely, extended homogeneous learning increases students’ cognitive load and diminishes their interest in studies, gradually leading to learner fatigue. To mitigate this, VS-based instructional tasks can be fragmented to prevent cognitive overload, with difficulty levels dynamically adjusted according to individual competencies. Developing a diverse library of virtual scenarios maintains novelty and engagement, avoiding monotony from repetitive simplistic content. Research indicates that physical side effects are associated with usage duration, visual design, and student operating behaviors (30), and these adverse effects could be mitigated through measures such as controlling session lengths, upgrading device capabilities, and optimizing technical parameters. Additionally, insufficient training or limited understanding of VS technology can result in operational frustration; thus, students without prior VS experience should receive appropriate pre-training before practice.

Due to its characteristics of interactive, ease of use, and entertaining, the advantages of VS in educational effectiveness have been extensively validated by prior studies (7). Human–machine interaction enables students to receive immediate operational feedback. Based on this feedback, students can reflect on their actions, adjust nursing practices, and internalize learning outcomes (25). Unlike traditional clinical practice, VS teaching platform allows students to repeatedly practice skills within specific clinical scenarios until mastery is achieved, thereby fostering advanced psychomotor abilities and emotional domain learning (11). The entertaining nature of VS enhances student engagement, strengthens motivation for self-directed learning, stimulates proactive problem-solving skills, and contributes to cultivating critical thinking capabilities.

The primary limitation of VS teaching lies in its lack of realism (31). Virtual patients, when interacting with students, are confined to predefined scenarios and fixed operational scripts within the system. They lack the ability to provide authentic patient feedback, and students cannot experience the genuine emotional connection of interacting with real patients. This limitation hinders the development of communication skills and humanistic care capabilities in medical education. To address these shortcomings, VS can be enhanced through hardware and software optimization. On the hardware front, improvements in resolution and refresh rates are essential, while software advancements should focus on elevating graphics rendering quality and incorporating haptic feedback mechanisms. These technical enhancements would create more lifelike simulation environments, thereby better facilitating the cultivation of clinical communication proficiency and empathetic patient care skills. The linguistic and cultural discrepancies inherent in VS environments present challenges for students. This issue is particularly pronounced among learners whose native language is not English or those with limited English proficiency, as English-dominated virtual contexts exacerbate their cognitive load. Research study by Tjoflat (32) corroborated that linguistic-cultural mismatches amplified psychological stress and obstruct effective learning. Consequently, integrating multilingual interface options within VS systems becomes essential to accommodate students from diverse linguistic and cultural backgrounds.

In response to the limitations of VS technology and challenges encountered by students in VS-based instruction, learners have expressed expectations for future VS device improvements. Considering that some students lack VS experience, simplifying operations and enhancing user-friendliness would facilitate quicker adaptation to VS teaching environments. Student feedback also highlighted insufficient scenario diversity in current VS applications, which fails to reflect the complexity of clinical contexts. It is recommended that subsequent VS software development prioritize incorporating comprehensive clinical scenarios and establishing multilingual case libraries to accommodate students from varied linguistic and cultural backgrounds. The high cost of VS equipment poses significant barriers to its adoption in medical education and practice. However, rapid technological advancements in recent years have gradually reduced VS hardware expenses, positioning VS as an increasingly accessible, educationally effective, and practical tool (28). Implementing VS technology holds potential to equalize practical teaching resources and provide technical support for equitable medical education.

Among the 17 included research studies, 10 utilized computer-based simulation platforms, while seven adopted highly immersive VR technologies, such as VR headsets, smart glasses, or haptic simulators. Both types of VS platforms greatly captured students’ interest and generally provided them with a positive learning experience. Students perceived that the drawbacks of these two VS teaching platforms differed in their focus: the shortcomings of the computer-based simulation platform mainly lay in the insufficient timeliness of feedback within the computer program (9); Jeon (33), whereas the limitations of the VR platform centered on the physiological discomfort caused by VR glasses or headsets, such as dizziness and nausea (19, 34). The differences in the limitations between the two platforms primarily stem from their respective technical constraints, suggesting that future software and hardware development should prioritize addressing these shortcomings at the technical level. On the pedagogical front, instructors can mitigate the lag in feedback by providing supplementary “immediate verbal feedback.” Concurrently, physiological discomfort among students can be alleviated by reasonably controlling the duration of VR training sessions.

### 6.1 Strengthens and limitations

The experiences of nursing students participating in VS teaching have received increasing attention, yet no research studies have synthesized these experiences to date. This research study integrates the VS teaching experiences of students from diverse backgrounds across different VS devices, providing a comprehensive description of students’ emotional experiences in VS-based instruction. This analysis further elucidates the challenges encountered and needs expressed by students during VS-based learning, offering a theoretical foundation for future improvements in VS teaching. However, our study has limitations. Due to language constraints, only Chinese and English databases were searched, potentially omitting gray literature. Cultural backgrounds and values vary across different countries, leading to distinct emphases on the cultivation of students’ competencies in VS teaching. The 17 studies included in this research were predominantly from Asian and European countries, and the language limitation has resulted in a deficiency in the adaptability of the study’s conclusions to multicultural contexts. Despite this, the included research studies originate from eight countries, allowing for a profound understanding of nursing students’ requirements for VS teaching through systematic integration.

### 6.2 Implications on future research and practice

This study reveals the limitations of emotional interaction in VS technology for nursing education. Future research needs to explore multimodal feedback mechanisms to enhance the realism of virtual patients. Meanwhile, the synthesis highlights a lack of cultural diversity in existing VS teaching scenarios. Developing multilingual case libraries and incorporating clinical practice standards from diverse countries are recommended. To reduce adaptation difficulties for students, future designs should optimize VS interfaces based on Cognitive Load Theory.

## 7 Conclusion

This review summarizes the experiences of nursing students with VS teaching, revealing their emotional responses to this approach, as well as the challenges and needs they encounter within such instructional contexts. Although the advantages of VS technology in enhancing students’ clinical practice skills have been well-documented, the future development of VR devices should prioritize the simplification of interactive design to reduce operational complexity. Exploring multimodal feedback mechanisms is crucial for improving the realism of virtual patients. Furthermore, developing case libraries that encompass diverse scenarios and multilingual support will enhance the richness and practicality of teaching.

## Data Availability

The original contributions presented in the study are included in the article/Supplementary material, further inquiries can be directed to the corresponding author.
