# Supplementary material for: Nursing students’ experiences in virtual simulation teaching: a meta-synthesis of qualitative research
Source: Front Public Health. 2026 Jun 22;14:1863553. doi: 10.3389/fpubh.2026.1863553 (PMC13333686; doi:10.3389/fpubh.2026.1863553)
Supplement: Supplementary file 1 [file Table_1.docx]

Supplementary Material 1: Pubmed search strategy

| Search | Query | Results |
| --- | --- | --- |
| #1 | Search: (nursing student*[Title/Abstract]) OR (students, nursing[MeSH Terms]) | 41359 |
| #2 | Search: (((virtual reality[MeSH Terms]) OR (virtual simulation[Title/Abstract])) OR (virtual reality[Title/Abstract])) OR (virtual reality simulation[Title/Abstract]) | 24224 |
| #3 | Search: ((((((((((((qualitative research[MeSH Terms])) OR (qualitative[Title/Abstract])) OR (phenomeno*[Title/Abstract])) OR (grounded theory[Title/Abstract])) OR (ethnography[Title/Abstract])) OR (experience*[Title/Abstract])) OR (feeling*[Title/Abstract])) OR (perception*[Title/Abstract])) OR (attitude*[Title/Abstract])) OR (perspective*[Title/Abstract])) OR (opinion*[Title/Abstract])) OR (emotion*[Title/Abstract]) | 3173755 |
| #1 AND #2 AND #3 | Search: (((((virtual reality[MeSH Terms]) OR (virtual simulation[Title/Abstract])) OR (virtual reality[Title/Abstract])) OR (virtual reality simulation[Title/Abstract])) AND (((((((((((((qualitative research[MeSH Terms])) OR (qualitative[Title/Abstract])) OR (phenomeno*[Title/Abstract])) OR (grounded theory[Title/Abstract])) OR (ethnography[Title/Abstract])) OR (experience*[Title/Abstract])) OR (feeling*[Title/Abstract])) OR (perception*[Title/Abstract])) OR (attitude*[Title/Abstract])) OR (perspective*[Title/Abstract])) OR (opinion*[Title/Abstract])) OR (emotion*[Title/Abstract]))) AND ((nursing student*[Title/Abstract]) OR (students, nursing[MeSH Terms])) | 272 |
